# Band Gap in Magnetic Insulators from a Charge Transition Level Approach

**DOI:** 10.1021/acs.jctc.0c00134

**Published:** 2020-05-19

**Authors:** Luis A. Cipriano, Giovanni Di Liberto, Sergio Tosoni, Gianfranco Pacchioni

**Affiliations:** Dipartimento di Scienza dei Materiali, Università di Milano—Bicocca, via R. Cozzi 55, 20125 Milano, Italy

## Abstract

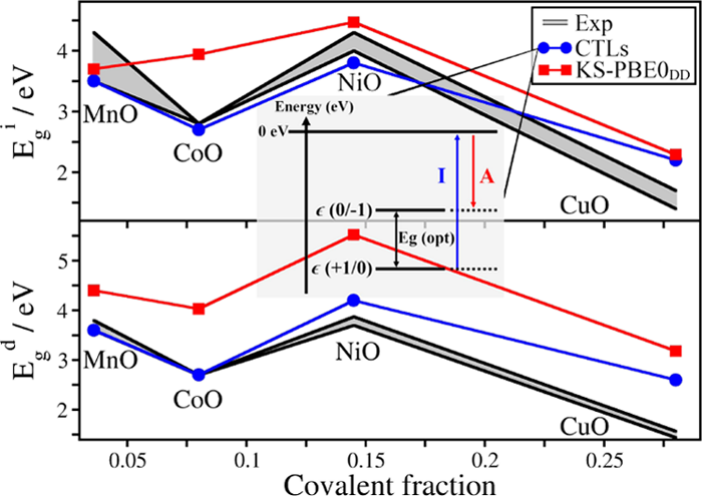

The theoretical description of the electronic structure of magnetic insulators and, in particular, of transition-metal oxides (TMOs), MnO, FeO, CoO, NiO, and CuO, poses several problems due to their highly correlated nature. Particularly challenging is the determination of the band gap. The most widely used approach is based on density functional theory (DFT) Kohn–Sham energy levels using self-interaction-corrected functionals (such as hybrid functionals). Here, we present a different approach based on the assumption that the band gap in some TMOs can have a partial Mott–Hubbard character and can be defined as the energy associated with the process M^*m*+^(3d*^n^*) + M^*m*+^(3d*^n^*) → M^(*m*+1)+^(3d^*n*–1^) + M^(*m*–1)+^(3d^*n*+1^). The band gap is thus associated with the removal (ionization potential, *I*) and addition (electron affinity, *A*) of one electron to an ion of the lattice. In fact, due to the hybridization of metal with ligand orbitals, these energy contributions are not purely atomic in nature. *I* and *A* can be computed accurately using the charge transition level (CTL) scheme. This procedure is based on the calculation of energy levels of charged states and goes beyond the approximations inherent to the Kohn–Sham (KS) approach. The novel and relevant aspect of this work is the extension of CTLs from the domain of point defects to a bulk property such as the band gap. The results show that the calculation based on CTLs provides band gaps in better agreement with experiments than the KS approach, with direct insight into the nature of the gap in these complex systems.

## 1 Introduction

Density functional theory (DFT) is commonly used to study the electronic structure of solids.^1^ One of the fundamental properties of a material is the band gap, which determines the optical, electrical, and chemical properties. The band gap calculation with DFT is well grounded, and it can be approximated by means of the analysis of the position of the Kohn–Sham (KS) energy levels.^1−5^ The accurate estimation of the band gap is challenging since KS energy levels are evaluated at the system’s ground state and because of the well-known problem arising from the choice of the proper DFT functional. The calculation of the band gap is usually based on the analysis of the position of the Kohn–Sham (KS) energy levels,^2^ despite the fact that DFT is a ground-state theory and that Kohn–Sham orbital energies provide, in principle, just a crude approximation of the band gap. The measurement of the band gap is also problematic, as it implies to excite one electron from the valence band (VB) to the conduction band (CB) (optical transition) or to remove or add electrons to the system as in photoelectron spectroscopies, causing important electronic and geometrical relaxations that follow the ionization process. In the first case, the measures are affected by the formation of excitons; in the second case, it is often difficult to distinguish initial- from final-state effects.^6^ Nevertheless, Kohn–Sham band gaps are widely and universally used due to their simplicity and rapid calculation.

KS-DFT in the local density (LDA) or generalized gradient (GGA) approximation is known to produce band gaps that are too small due to the self-interaction error.^7^ A more accurate description of the KS band gap can be obtained using the hybrid functionals^8−11^ or the DFT + *U* approach.^12,13^ However, also hybrid functionals and the DFT + *U* method are not free from limitations. In hybrid functionals, the exchange energy is constructed including a portion α of exact Fock exchange, where α depends on the formulation used. For instance, α = 0.2 is used in the popular B3LYP method,^8,9,14^ while α = 0.25 is adopted in the better grounded PBE0^10,15^ and HSE06^11,16^ functionals. Since the value of α can be varied from 0 (pure GGA approach) to 1 (pure Fock exchange), sometimes this is tuned in an empirical way to obtain a band gap that fits with the experimental results. Clearly, this introduces a given level of empiricism. The same applies to the determination of the U value in LDA + *U* or GGA + *U* approaches. While methods to determine this from first principles have been proposed,^17^ the *U* term is usually derived for each system based on a comparison of measured and computed properties (not only the band gap but also magnetic properties, chemical reactivity, etc.). Once more, this makes the method rather empirical and system-dependent. The dependence of the results on the choice of the α or *U* parameters represents a severe limitation in the predicting value of DFT calculations of the band gap of insulating and semiconducting materials.

Despite all of these problems, KS-DFT is still widely used to estimate the band gap of solid systems. These include also a class of highly correlated solids, such as transition-metal oxides and, in particular, oxides of the elements at the right-hand side of the first transition-metal row (Mn, Fe, Co, Ni, Cu). These oxides are magnetic insulators, i.e., they exhibit insulating character, high-spin configurations, and often antiferromagnetic ordering, i.e., nearby metal cations display opposite spin. Their electronic structure is characterized by a predominant contribution of metal’s d-orbitals to both the valence and conduction bands. Differently from the charge transfer oxide insulators, where electronic excitations correspond to an actual transfer from O(2p) orbitals to metal’s d-orbitals, electronic excitations in these materials are rather described as an electron hopping from one metal center to the next. Their description in terms of band model raises fundamental questions, and for this reason, they are classified as magnetic insulators or even as Mott–Hubbard insulators in the intermediate regime.^18^ In this class of materials, narrow bands are formed due to the very localized nature of the 3d orbitals of late transition metals or the f orbitals of rare-earth elements. This is also the reason why these are often referred to as highly correlated solids. The treatment of complex oxides requires to go beyond the analysis of KS levels,^19^ and more accurate as well as computationally demanding approaches have been proposed, such as the GW^20^ or the dynamical mean-field theory (DMFT) methods.^21^

MnO, FeO, CoO, NiO, and CuO are characterized by the presence of atomic-like cation’s 3d orbitals occupied by a number of electrons that go from 5 (Mn) to 9 (Cu). This leads to the presence of occupied and unoccupied metal 3d states and a permanent magnetic moment (all of these systems are antiferromagnets at the ground state). A very simplified model to describe conductivity in these systems was proposed by Hubbard in 1963.^22^ In the Hubbard model, the electron mobility is due to the excitation of one electron from a metal cation to an adjacent neighboring cation (hopping) according to the following equation (where the oxidation state of the cation is that of the TM monoxides)
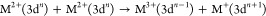
1According to this oversimplified view, the band gap (*E*_g_) of the system is approximated as *E*_g_ = *U* = *I* – *A*, where *I* and *A* are the ionization potential [M^2+^(3d*^n^*) → M^3+^(3d^*n*–1^) + e^–^] and the electron affinity [M^2+^(3d*^n^*) + e^–^ → M^+^(3d^*n*+1^)] of an M^2+^(3d*^n^*) ion, respectively. Thus, the band gap can be approximated as the difference between two atomic total energies, that of the metal cation with one electron removed (*I*) and with one electron added (*A*). In fact, solid-state effects largely contribute to modify the band gap from what is predicted based on this model. These have been included in the classical Zaanen, Sawatzky, and Allen (ZSA) theory.^23^ The ZSA model of insulating TM compounds is based on a comparison of the on-site correlation energy, *U*_dd_, and the charge transfer energy, Δ. When *U*_dd_ < Δ, the energy gap *E*_g_ is determined by *U*_dd_ (*E*_g_ ≈ *U*_dd_) corresponding to the transition 3d*^n^* + 3d*^n^* → 3d^*n*–1^ + 3d^*n*+1^ giving rise to a Mott–Hubbard insulator. When *U*_dd_ > Δ, the charge transfer energy Δ, corresponding to the transition 3d*^n^* → 3d^*n*+1^L (where L indicates a hole on a ligand), determines the gap (*E*_g_ ≈ Δ) and the system is classified as a charge transfer insulator. In the ZSA model, the relative weight of the two terms is empirically defined, while here it is the result of the full ab initio determination of the final wave functions.

The present work stems from the idea that the late TM oxides have partial Mott–Hubbard insulator character. In the DFT framework, the band model in connection with standard functionals, such as GGA, fails to properly describe these materials. For instance, in GGA, CoO is classified as a metal,^24−27^ contrary to every evidence.^28−30^ Similar problems have been found for NiO with the LDA method,^31−33^ whereas NiO is known to be a charge transfer insulator.^23,34,35^ In fact, the situation improves considerably if one uses self-interaction-corrected functionals, in particular hybrid functionals, and recently, various studies have been reported showing a good performance of these functionals in determining the band gap of TM monoxides.^25−27,36^ However, in some cases, the deviation from experimental values is still significant. Moreover, this conclusion is based on the KS energies, which once more implies the use of a ground-state theory to describe an excited-state problem.

To overcome these limitations in the TM monoxides, advanced approaches such as dynamic mean-field theory,^29,35,37^ random phase approximation,^38,39^ and GW^40−45^ have been applied to fill the gap between the KS band gaps and the experimental ones. However, in this paper, our purpose is to suggest an alternative approach to the calculation of the band gap in these systems within the frame of DFT. We show that our approach is relatively well behaved to the KS band gaps for the TM monoxides. A first example of this approach has been recently reported for the study of CuWO_4_, also a magnetic insulator.^46^ The idea is the following. The ground-state properties of the TM oxides are obtained with a hybrid functional to provide a good ground-state density and electronic structure. To go beyond the approximations inherent to KS-DFT, we have computed the band gap starting from the consideration that they are all characterized by rather localized 3d orbitals. Then, we used the charge transition level (CTL)^47−51^ scheme normally adopted to compute electronic transitions for defects in insulators, to estimate the band gap of the material. This procedure, based on adding or removing one electron to/from the system, and not on one-electron levels of the ground state, is thus an alternative and better grounded approach and can be used to provide a validation of KS-DFT band gaps.^46^ Thus, the central idea is to determine the energy associated with the process described in eq 1 as a measure of the band gap and to compare this with KS band gaps and with the experimental values.

Since we have seen above that hybrid functionals depend on the value of the α term used, we adopted a strategy where the optimal Fock exchange fraction α is determined in a self-consistent way from an *ab initio* approach. The starting point is that α is inversely proportional to the dielectric constant of a material.^52−54^ The use of dielectric-dependent hybrid functionals has shown an improvement in the description of several materials, including inorganic^26,55^ and organic compounds;^56^ recently, it has also been successfully applied to the same class of solids of interest in this paper, i.e., MnO, FeO, CoO, and NiO.^27^ It should be mentioned, however, that while dielectric-dependent functionals improve the description of selected solids, this is not necessarily a universal way to solve the problems of hybrid functionals. In fact, in a recent study on 24 semiconducting oxides and 24 layered materials, we have shown unambiguously that there is no systematic improvement in the use of dielectric-dependent functionals and that this works better only in some cases.^57^

This paper is organized as follows. In Section 2, we describe in detail the computational procedure adopted, introducing the concept of dielectric-dependent hybrid functionals, the supercells, and the basis sets used and providing a brief explanation of the procedure based on the calculation of the charge transition levels. The results are presented in Section 3 and have been divided into six subsections: Cu_2_O (test case), MnO, FeO, CoO, NiO, and CuO. In each of these subsections, the results from KS-DFT are compared to those of the CTLs. Some general discussions and conclusions are provided in Section 4.

## 2 Methods

### 2.1 Dielectric-Dependent Hybrid Functionals

It can be shown that the fraction α of exact exchange in hybrid functionals is related to the inverse of the static dielectric constant ε_∞_^52−54^
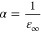
2where ε_∞_ can be evaluated from the independent particle approximation^58^ and the random phase approximation.^59^ The nonlocal contributions to the dielectric response can be explicitly taken into account by determining the Kohn–Sham orbitals in a self-consistent way, using the coupled-perturbed Kohn–Sham (CPKS) method.^60−62^ For some materials, this approach gives computed dielectric constants in agreement with the experiment, and sometimes the accuracy is comparable to that of self-consistent GW calculations.^63^ The self-consistent hybrid dielectric-dependent (DD) approach has been used to study oxides,^55,57,64−68^ nitrides,^69,70^ magnetic insulators,^26,27^ etc. An alternative model to the dielectric-dependent approach to fix the amount of nonlocal exchange and provide good band gaps is to design functionals that satisfy Koopmans’ condition, such as those based on a set of parameters suggested by Moussa et al.^71^ and later by Deák et al.^72^

We performed spin-polarized hybrid functional calculations using the PBE0 formulation^10,15^ with the fraction of exact Fock exchange calculated using the coupled-perturbed Kohn–Sham (CPKS)^60−62^ method, as implemented in CRYSTAL17 code.^73^ We refer to the functional based on PBE0, where α was self-consistently determined as PBE0_DD_. The self-consistent dielectric-dependent procedure to determine the optimal α has been done at the experimental geometry.^57^

For the sake of comparison, we repeated KS calculations with the screened hybrid functional HSE06 at the geometry optimized with PBE0. Similarly, we took the geometry and the self-consistently determined α from PBE0_DD_ and performed KS band gaps with HSE06; we refer to this latter functional as HSE06_DD_. The choice to rely on α as determined with PBE0_DD_ is due to the fact that the dielectric constant calculation with HSE is not implemented in the CRYSTAL code. Results with HSE06 and HSE06_DD_ are reported in Section 4.

### 2.2 Charge Transition Levels

It is possible to estimate the position of energy levels introduced by an isolated defect in the gap of a semiconductor by considering the charge transition level (CTL) approach.^47−51^ Here, the total energies of different electronic states are considered instead of one-electron KS energies. Usually, the transition level ϵ(*q*/*q*′) is defined as the Fermi level, referred to the top of the VB, for which the formation energies of defects in the charge states *q* = *q*′ + 1 and *q*′ are equal. Here, the CTLs can be derived on the basis of Janak’s theorem.^74^ This method is rather accurate when used in connection with hybrid functionals^53,75−80^ and allows us to circumvent the problem of the calculation of the total energy of charged supercells, which is not possible with the CRYSTAL code because of the interaction with the balancing background of charge.^81^

When calculating the charge transition levels, various supercells (from a few to more than 100 atoms) have been used to check the convergence of the data with supercell size. It is worth noting that, while the KS gap is substantially invariant with respect to the cell’s size, CTLs imply the creation of charged species, whose stability is largely influenced by their reciprocal distance. It is thus mandatory to check the convergence of the CTL gap with respect to the supercell’s size.

The energy gap of TM oxides has been estimated as the difference between the ionization potential (*I*) and the electron affinity (*A*) of the system (eq 1). The procedure starts from the ground-state electronic structure of the neutral oxide, by removing or adding one electron, forming the corresponding +1 and −1 charged states. Optical transition levels (ϵ^opt^) are estimated while keeping the atoms fixed in their fundamental state’s positions, while thermodynamic transition levels (ϵ^therm^) are calculated on the fully relaxed charged system (see Section S4 in the Supporting Information (SI)).

The formation energy ϵ^opt^ (*q*/*q*′) of these charge states has been obtained following the approach described in detail by Gallino et al.,^78^ where Janak’s theorem is used starting from this expression
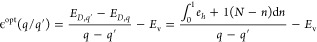
3where *E*_*D*,*q*′_ and *E*_*D*,*q*_ are the total energies of the defective systems with charge *q*′ and *q* = *q*′ + 1, respectively; *e*_*h*+1_(*N*) is the KS eigenvalue of the lowest unoccupied (*h* + 1) state of the *q* charged state (*N* electrons); and *E*_v_ is the valence band maximum of the neutral system. We see that eq 3 can be solved by performing the energy difference between defective systems. One possible way to evaluate it without using total energies of charged supercells is by using mean value theorem. After taking into account the mean value theorem for the integrals, it is possible to compute the formation energy ϵ^opt^(*q*/*q*′) of the charged states following eq 4, where the calculation of two eigenvalues is required instead of all of the eigenvalues of *n* between 0 and 1
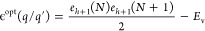
4Here, e_*h*+1_(*N*) and e_*h*+1_(*N* + 1) are the KS eigenvalues of the lowest unoccupied state (LUMO) in the charge state *q* and the highest occupied state (HOMO) in the charge state *q*′, respectively.

The ionization potential, *I*, can thus be obtained as
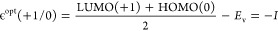
5while the electron affinity, *A*, can be written in terms of the corresponding transition levels
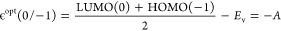
6Then, the optical energy gap, *E*_g_(opt), is calculated as the difference of ionization potential and electron affinity, which in turn is the difference between ϵ^opt^(0/–1) and ϵ^opt^(+1/0), determined with respect to the top of valence band in the host material
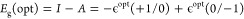
7Note that this definition of optical gap does not include exitonic effects and that, for this reason, this quantity is sometimes referred to as the fundamental gap. Optical transition levels (ϵ^opt^), as stated above, do not include relaxation effects and can be directly compared to the position of the band edges estimated from optical excitation.

The direct comparison between experimental excitation energies and calculated band gaps will be based on the optical (fundamental) gaps, i.e., keeping frozen the nuclei configurations. This assumption is based on the shorter time lapse associated with photon absorption/emission (femtoseconds) compared to atomic relaxation (picoseconds). On the other hand, it can be interesting to estimate also the effects related to the geometrical relaxation upon trapping of charged species.^78,82^ This information may allow us to rationalize the accuracy of KS-DFT gaps since this effect is generally neglected when looking at KS energy levels of the neutral system. Moreover, the estimate of relaxation energies provides additional information about the nonradiative decay of photoexcited electrons and holes.

The direct or indirect nature of the energy gap is indicated by i or d, *E*_g_^i^ or *E*_g_^d^, respectively. From CTLs, the direct band gaps have been computed taking the eigenvalues at the Γ point (HOMO and LUMO), while the indirect band gaps were computed taking the eigenvalues with the highest and lowest energies at other *k*-points. Finally, we have to mention that despite several attempts, we were not able to converge the CTL calculations for FeO, which therefore will be discussed only at the level of hybrid functional (see below).

A graphical sketch of the charge transition levels associated with the removal, ϵ^opt^(+1/0), and addition, ϵ^opt^(0/–1), of one electron is shown in Figure S2 in the SI.

### 2.3 Basis Sets, Supercells, Tolerances

Calculations have been done with all-electron Alhrichs-type basis sets (Pob-TZVP^83^) for Mn, Fe, Co, Ni, Cu, and O atoms. In selected cases, the results have been checked versus basis set type and quality (see Table S1 in the SI). Based on this comparison, we concluded that the Pob-TZVP basis set provides a good accuracy; for instance, on the lattice parameters of the cubic unit cells.^84^

The cutoff value for coulomb and exchange integrals in self-consistent field (SCF) calculations was 10^–7^ for coulomb overlap tolerance, coulomb penetration tolerance, exchange overlap tolerance, and exchange pseudo-overlap in direct space and 10^–14^ for exchange pseudo-overlap in reciprocal space. The SCF calculation was considered converged when the difference in energy between two subsequent cycles was lower than 10^–8^ atomic units (au). The sampling of the reciprocal space was adapted to the size of the supercell: a shrinking factor of 8 in the Pack–Monkhorst scheme was adopted for the 2 × 1 × 1 unit cells (these cells have two TM oxides (Mn, Fe, Co, Ni, and Cu) and two O atoms along the [111] direction and allowed us to compute the antiferromagnetic (AFM) and ferromagnetic (FM) solutions in the TM monoxides) and subsequently reduced to 2 when working on larger supercells.

The simulated cells have been fully optimized (both atomic coordinates and lattice parameters), and simulations were considered converged with threshold values of 0.0003 and 0.0045 au for the root-mean-square (rms) and maximum absolute values of the gradient, respectively, and of 0.0012 and 0.0018 au for rms and maximum displacements, respectively. The ferromagnetic (FM) and a few possible antiferromagnetic (AFM) configurations were considered for each oxide. Only the most stable magnetic structure will be described and used for further investigations.

When evaluating charge transition levels (see below), it is more difficult to reach convergence, especially with large supercells. In these cases, the tolerance on the total energy was changed to 10^–7^ au. The truncation criteria for the two-electron integrals were unchanged.

The density of states (DOS) curves for the 2 × 1 × 1 unit cells (see Figure S1 in the SI) were determined with shrinking factor 10 for reciprocal space Pack–Monkhorst net and also for reciprocal space Gilat net.

## 3 Results

### 3.1 Test Case: Cu_2_O

The first case is that of Cu_2_O, a nonmagnetic oxide. Nevertheless, it has a narrow Cu 3d band and can be used to verify if the procedure adopted for the calculation of the band gap using the CTLs is sufficiently accurate.

Cu_2_O crystallizes in the *Pn*3̅*m* [224] point group; the lattice parameter is 4.2685 Å.^85^ The first set of calculations consists of the determination of the optimized ground-state properties using the standard PBE0 functional and the corresponding dielectric-dependent version, PBE0_DD_.

For this system, a self-consistent dielectric-dependent α = 0.245 was found, corresponding to a dielectric constant 4.09, Table 1. This is practically the same dielectric constant found in PBE0 for the optimized geometry. Therefore, the two approaches provide very similar direct KS band gaps: *E*_g_^d^ (PBE0) = 2.84 eV and *E*_g_^d^ (PBE0_DD_) = 2.79 eV (Table 1).

**Table 1:** KS-DFT Direct Band Gap (*E*_g_^d^ in Electronvolts) and Dielectric Constant for Cu_2_O

Cu_2_O	*E*_g_^d^ (eV)	ε_∞_
PBE0 (α = 0.25)	2.84	4.06
PBE0_DD_ (α = 1/sc – ε_∞_)	2.79	4.09
exp.	2.17–2.62^a^	6.46^b^

a Optical absorption.

b See ref (86).

All calculations give a dielectric constant for Cu_2_O, which is about 40% smaller than the experimental one, 6.46.^86^ Similar values are also obtained by determining the optimal α by taking the experimental dielectric constant (PBE0_exp_), as reported in Table S2.

Experimentally, optical absorption studies report values of the direct band gap in the range of 2.17–2.62 eV;^87^ thus the PBE0 and PBE0_DD_ values, about 2.8 eV, are close to the upper limit of the experimental measurements. As we already mentioned, optical absorption experiments usually provide a lower bound to the real band gap due to the presence of excitons.

Now we consider the band gaps in Cu_2_O, as obtained using the approach described above and based on the CTLs at the level of PBE0_DD_. The results have been checked versus supercell size using cells containing 6, 12, 24, 48, 72, and 108 atoms (Table 2).

**Table 2:** Direct (*E*_g_^d^) and Indirect (*E*_g_^i^) Band Gap (in Electronvolts) of Cu_2_O for Supercells of Increasing Size Computed According to the CTL Method

cell size	1 × 1 × 1	2 × 1 × 1	2 × 2 × 1	2 × 2 × 2	3 × 2 × 2	3 × 3 × 2
no. of atoms	6	12	24	48	72	108
*E*_g_^d^	0.14	1.52	2.02	2.63	2.47	2.55
*E*_g_^i^	0.05	2.52	2.70	2.99	3.19	3.10

We discuss first the nature of the transition. Here, the ionization involves a Cu^+^(3d^10^) ion, which formally becomes Cu^2+^(3d^9^). On the other hand, the addition of one electron to the supercell results in a delocalized electron that occupies the Cu 4sp band.

**Figure 1:**
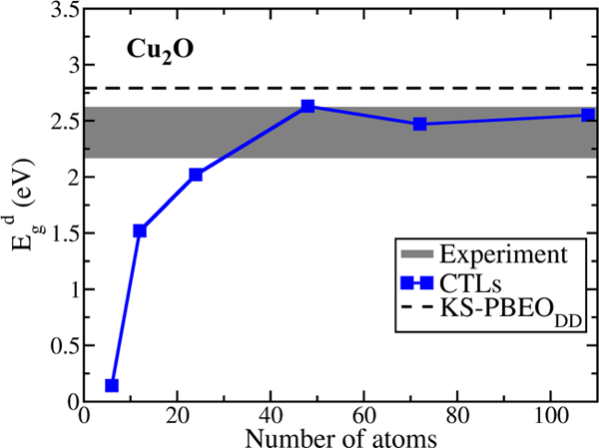
Direct band gaps of Cu_2_O computed with the CTL approach as a function of the supercell size. The black dashed line represents the direct KS-PBE0_DD_ band gap, and the gray interval represents the range of experimental values.

Not surprisingly, the six-atom unit cell is too small and cannot be used to obtain reasonable values of the gap (calculated neglecting structural relaxation, Table 2). Even the 12-atom cell is not sufficient, while the results tend to become stable (*E*_g_ variations within 0.2 eV) with larger supercells (Figure 1). If we look at the largest cell considered, 3 × 3 × 2 (108 atoms), it gives a direct band gap of 2.55 eV, in excellent agreement with the experimental estimates (2.17–2.62 eV; see also Figure 1).

We have then relaxed the charge cells to evaluate the impact of structural relaxation, finding for the largest supercells small relaxation effects, around 0.11 eV (Figure S3) in the SI.

The gap values computed with the CTL approach are quite similar to those obtained at the PBE0_DD_ KS level (see Tables 1 and 2 and Figure 1). In particular, the direct band gap obtained with CTLs (2.55 eV) is within the experimental range (2.17–2.62 eV), and it is slightly closer to experiment than the KS one (2.79 eV). On the one hand, this validates the procedure followed; on the other hand, it provides a justification for the use of the KS method to estimate the band gap of Cu_2_O. Finally, we note that the prediction of a direct nature of the band gap is found using both the KS and CTL approaches.

Having demonstrated that the procedure works for the simple case of the nonmagnetic Cu_2_O oxide, we now move to the more complex late TM oxides.

### 3.2 MnO

MnO has an *Fm*3̅*m* space group (lattice parameter *a* = 4.445 Å).^84^ In the ground state, due to the octahedral crystal field, the Mn^2+^ ion has a (*t*_2g_)^3^(*e*_g_)^2^ configuration, with five unpaired electrons per Mn ion. We compute a spin population of 4.82 μ_B_ per Mn ion, very close to the nominal value, indicating a high level of ionicity of this oxide (the covalent contribution is less than 4%, using the spin population as a measure). At the PBE0 level, we found that the AFM state is 0.16 eV lower in energy than the FM one. The AFM configuration will be used in the following for the study of the ground-state properties of MnO.

The lattice constants of MnO have been fully optimized, along with the atomic coordinates. At the PBE0 KS level, MnO exhibits an indirect band gap of 3.93 eV and a direct gap of 4.62 eV (Table 3). The dielectric constant is ε_∞_ = 4.26.

**Table 3:** KS-DFT Indirect (*E*_g_^i^) and Direct (*E*_g_^d^) Band Gaps (in Electronvolts), Magnetic Moment (μ_B_/atom), and Dielectric Constant for MnO

MnO	*E*_g_^i^ (eV)	*E*_g_^d^ (eV)	*M* (μ_B_/atom)	ε_∞_
PBE0 (α = 0.25)	3.93	4.62	4.82	4.26
PBE0_DD_ (α = 1/sc – ε_∞_)	3.70	4.40	4.81	4.34
exp.			4.58^a^	4.95^b^
XAS + XES	4.1^c^			
PES + BIS	3.9 ± 0.4^d^			
optical absorption		3.6–3.8^e^		

a See ref (89).

b See ref (88).

c See ref (29).

d See ref (90).

e See ref (91).

The next step consists of the calculation of MnO using the dielectric-dependent version of the PBE0 functional. Determining ε_∞_ in a self-consistent way leads to ε_∞_ = 4.34 corresponding to an α value of 0.230; this is quite close to α = 0.25 in PBE0. It is not surprising that the PBE0_DD_ indirect band gap, *E*_g_^i^ = 3.70 eV, is slightly smaller than at the PBE0 one. The direct gap, *E*_g_^d^ = 4.40 eV, is also similar to PBE0, and the magnetization is the same (Table 3). The computed dielectric constant, 4.26–4.34, is sufficiently close to the experimental one, 4.95.^88^

Experimentally, band gap values in the range 3.6–4.1 eV have been reported (Table 3) with gaps derived from optical absorption measurements smaller than those from photoemission.

**Figure 2:**
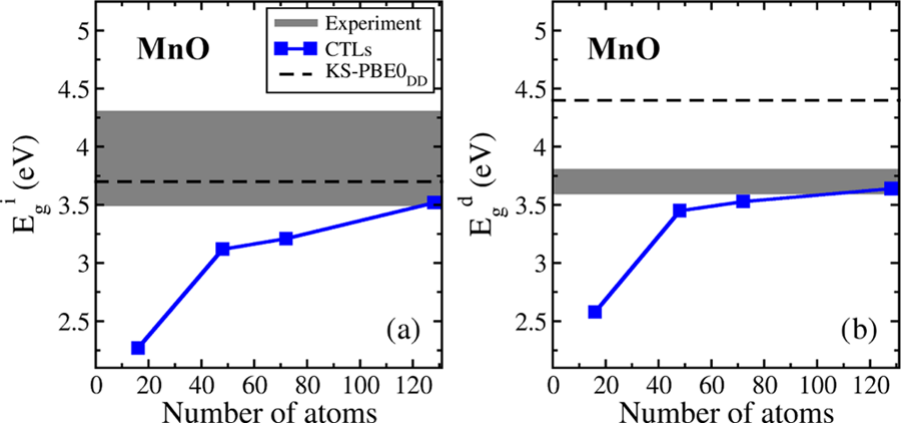
(a) Indirect and (b) direct band gaps of MnO computed with the CTL approach as a function of supercell size. The black dashed lines represent the indirect and direct KS-PBE0_DD_ band gap, and the gray interval represents the range of experimental values.

The next step consists of the determination of the band gap of MnO using the procedure based on the CTLs using supercells of increasing dimensions (Table 4 and Figure 2). We first discuss the nature of the electronic state obtained by adding or removing one electron to/from the supercell. When we compute the positively charged supercell with PBE0_DD_, the spin population of a Mn ion goes from 4.81 μ_B_/atom, ground state, to 3.91 μ_B_/atom, with a reduction of 0.9 unpaired electrons. The hole is fully localized on a single Mn ion, which formally changes its state from Mn^2+^(3d^5^) to Mn^3+^(3d^4^). The addition of an excess electron at the same level of calculation, however, results in different situations depending on the supercell used. With the largest supercell, we find a reduced Mn ion with a spin population of 4.29 μ_B_/atom, and a localization of about 0.5 excess electrons, which formally goes from Mn^2+^(3d^5^) to Mn^+^(3d^6^). However, the 4 × 3 × 3 supercell (as well as any smaller one) gives a completely delocalized solution for the excess electron. For each calculation, we carefully looked for other localized/delocalized solutions by starting from different initial guesses of the electron density. We cannot exclude that other solutions could be obtained.

**Table 4:** Indirect (*E*_g_^i^) and Direct (*E*_g_^d^) Band Gap (in Electronvolts) of MnO for Supercells of Increasing Size Computed According to the CTL Method

cell size	2 × 2 × 2	4 × 3 × 2	4 × 3 × 3	4 × 4 × 4
no. of atoms	16	48	72	128
*E*_g_^i^	2.27	3.12	3.21	3.52
*E*_g_^d^	2.58	3.45	3.53	3.64

The smallest supercell for which it was possible to achieve convergence on single-point calculations for the charged systems is the 2 × 2 × 2 one. The results become stable starting from the 4 × 3 × 2 supercell (48 atoms, Figure 2). The indirect band gap increases regularly by increasing the supercell size, and it becomes *E*_g_^i^ = 3.52 eV with the 4 × 4 × 4 supercell (128 atoms). These values are only slightly smaller than the KS indirect band gap computed at the PBE0, 3.91 eV, or at the PBE0_DD_, 3.70 eV, levels. In fact, this could be partly fortuitous as the results are not yet fully converged with respect to the supercell size (Table 4 and Figure 2). Unfortunately, going to even larger supercells becomes too demanding in terms of computational resources required. Nevertheless, we think that the similarity between the CTL indirect band gaps and the KS-DFT one is clear. This provides a validation of the band gap for a magnetic insulator computed at the KS-DFT level. Indeed, the indirect nature of the band gap, which results from KS calculations as well as from GW approaches,^40^ is confirmed by the present CTL methodology. Differently from the indirect gap, the direct gap estimate significantly improves, moving from 4.4–4.6 eV (KS-DFT) to 3.6 eV (CTLs), to be compared to the experimental range (3.6–3.8 eV). As for the previous cases, the relaxation effects on large charged cells are small, around 0.15 eV (Figure S3).

### 3.3 FeO

We discuss here FeO, although we can report only partial results on this material due to severe convergence problems. Several attempts and efforts have been made to overcome these problems, mostly related to the calculation of the dielectric constant of the material and of the charged supercells required to obtain the band gap with the CTL approach. Therefore, only KS-DFT results are reported.

FeO has an *Fm*3̅*m* point group (a = 4.332 Å).^84^ The AFM state is 0.24 eV lower in energy than the FM one. At the PBE0 level and with full optimization of the unit cell, the gaps are *E*_g_^i^ = 2.04 eV and *E*_g_^d^ = 2.11 eV, with the magnetization of 3.73 μ_B_/atom. Due to problems in the calculation of the dielectric constant, we cannot report PBE0_DD_ results. Then, we also determined the ground-state properties using the average experimental dielectric constant, (10.17, computed from refs (92, 93)) finding much smaller gap values (around 0.8 eV) and a similar magnetization, as reported in Tables 5 and S2.

**Table 5:** KS-DFT Indirect (*E*_g_^i^) and Direct (*E*_g_^d^) Band Gaps (in Electronvolts) and Magnetic Moment (μ_B_/atom) for FeO

FeO	*E*_g_^i^ (eV)	*E*_g_^d^ (eV)	*M* (μ_B_/atom)
PBE0 (α = 0.25)	2.04	2.11	3.73
PBE0_exp_ (α = 1/ε_exp_)	1.22	1.29	3.72
optical absorption		2.40^a^	3.32^b^, 4.20^c^

a See ref (95).

b See ref (96).

c See ref (97).

These data can be compared to the literature at the same level of theory (PBE0), but with plane-wave codes. A significant difference is found with the recent paper of Liu et al.,^27^ where *E*_g_^i^ = 3.02 eV and *E*_g_^d^ = 3.42 eV are reported. In another PBE0 study by Tran et al.,^24^ the indirect and direct band gaps are 1.20 and 1.60 eV, respectively. The large differences found in the KS-DFT band gap for FeO show that this system is particularly delicate and is very challenging for DFT-based electronic structure approaches. Alfredsson et al.^94^ studied FeO using hybrid functionals and the CRYSTAL code; they found that about 30–60% of Fock exchange is needed to correctly reproduce the electronic structure of this material. We also noted that Skone et al.^26^ computed the dielectric constants and band gaps for MnO, CoO, and NiO applying the self-consistent dielectric-dependent method with PBE0 functional in combination with the CRYSTAL code, but they did not report the values for FeO, probably due to the same kind of problems discussed here.

### 3.4 CoO

In CoO (*Fm*3̅*m* space group; lattice parameter, *a* = 4.260 Å),^84^ the Co^2+^ ion has a (*t*_2g_)^5^(*e*_g_)^2^ configuration and three unpaired electrons. We compute a magnetization of 2.76 μ_B_ per Co ion, close to the nominal value. The covalent contribution is thus 8%, slightly higher than that in MnO. At the PBE0 level, the AFM state is 0.14 eV lower in energy than the FM one, and will thus be used in the following. On a fully optimized structure at the PBE0 level, CoO exhibits an indirect KS band gap *E*_g_^i^ = 4.71 eV and a direct gap *E*_g_^d^ = 4.87 eV (Table 6). The dielectric constant is ε_∞_ = 4.54.

**Table 6:** KS-DFT Indirect (*E*_g_^i^) and Direct (*E*_g_^d^) Band Gaps (in Electronvolts), Magnetic Moment (μ_B_/atom), and Dielectric Constant for CoO

CoO	*E*_g_^i^ (eV)	*E*_g_^d^ (eV)	*M* (μ_B_/atom)	ε_∞_
PBE0 (α = 0.25)	4.71	4.87	2.76	4.54
PBE0_DD_ (α = 1/sc – ε_∞_)	3.93	4.03	2.73	4.92
exp.			3.35^a^	5.35^b^
XAS + XES	2.6^c^			
PES + BIS	2.5 ± 0.3^d^			
optical absorption		2.7^e^		

a See ref (99).

b See ref (98).

c See ref (29).

d See ref (28).

e See ref (30).

Using the PBE0_DD_ functional, we find ε_∞_ = 4.92 and a corresponding α = 0.203. The indirect KS band gap becomes *E*_g_^i^ = 3.93 eV, and the direct one *E*_g_^d^ = 4.03 eV (Table 6). The change in magnetization with respect to PBE0, 0.03 μ_B_/atom, is negligible. In Table S2, we also report the corresponding PBE0_exp_ estimates, obtained from the experimental dielectric constant 5.35,^98^ only slightly higher than the PBE0_DD_ computed one.

The experimental direct and indirect band gaps derived from optical absorption or photoemission measurements are very close to each other, which is consistent with the KS results (Table 6). However, the computed KS values span in the range of 3.93–4.87 eV, and they are considerably larger than the experimentally reported band gaps, which are in the range of 2.5–2.7 eV (Table 6).

In Table 7, we report the band gaps computed according to the CTL procedure starting, also in this case, from the PBE0_DD_ ground state. When we compute the positively charged supercell, we found a Co ion where the magnetization is reduced from 2.73 μ_B_/atom, ground state, to 1.90 μ_B_/atom, with a reduction of 0.83 unpaired electrons. Thus, the hole is localized on a single Co ion, which changes configuration from Co^2+^(3d^7^) to Co^3+^(3d^6^). The addition of an excess electron leads to one Co ion with magnetization 2.01 μ_B_/atom. In this case, the excess electron is fully localized, and the process corresponds to Co^2+^(3d^7^) + e^–^ → Co^+^(3d^8^).

**Table 7:** Indirect (*E*_g_^i^) and Direct (*E*_g_^d^) Band Gap (in Electronvolts) of CoO for Supercells of Increasing Size Computed According to the CTL Method

cell size	2 × 1 × 1	4 × 3 × 2	4 × 3 × 3	4 × 4 × 4
no. of atoms	4	48	72	128
*E*_g_^i^	0.08	2.84	3.13	2.66
*E*_g_^d^	0.30	2.96	3.20	2.71

**Figure 3:**
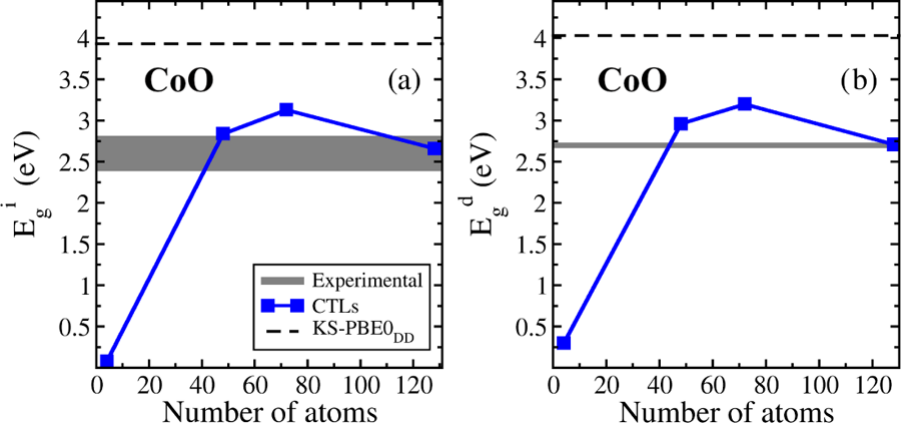
(a) Indirect and (b) direct band gaps of CoO computed with the CTL approach as a function of the supercell size. The black dashed lines represent the indirect and direct KS-PBE0_DD_ band gap, and the gray interval represents the range of experimental values.

The smallest supercell for which it was possible to achieve convergence for single-point calculations on the charged systems is the 2 × 1 × 1 one. However, the corresponding band gaps are still way off any convergence. The results become stable and comparable starting from the 4 × 3 × 2 supercell (48 atoms, Figure 3), even if some oscillations are present. *E*_g_^i^ becomes 2.66 eV in the 4 × 4 × 4 supercell, to be compared to 3.9–4.7 eV (KS-DFT), and is in very good agreement with the experimental estimates, 2.5–2.7 eV. Similarly, the direct band gap, *E*_g_^d^ = 2.71 eV, is consistent with the experiment, while the KS-DFT one is in the range of 4.0–4.9 eV. Even if some oscillations in the bang gap with CTLs are present, this shows that for CoO, going from the KS-DFT approach to the CTL one, there is a clear improvement of the results, leading to a band gap in much better agreement with the experiment, as we clearly observe in Figure 3. For this system, upon a full relaxation of the charged cell, we noted a significant structural relaxation following the polaron formation, around 0.61 eV (Figure S3 in the SI).

In conclusion, for CoO, we found that the CTL approach gives indirect and direct band gaps (2.6–2.7 eV) in much better agreement with the experiment (2.5–2.7 eV) than all of the KS-DFT calculations considered in this work. It is worth noting that this is a particularly challenging system since also other forms of hybrid functionals, such as the range-separated HSE06,^27^ overestimate the KS band gap of CoO with *E*_g_^i^ = 3.50 eV and *E*_g_^d^ = 4.29 eV, in line with the values reported in Table 6.

### 3.5 NiO

The lattice constant of NiO (*Fm*3̅*m* space group, *a* = 4.177 Å) is slightly shorter than that in CoO.^84^ The Ni^2+^ ion has a (*t*_2g_)^6^(*e*_g_)^2^ configuration and a triplet state. The magnetic moment is 1.71 μ_B_ per Ni ion, with a deviation of 14.5% from the nominal ionicity. Thus, the covalent character increases as we move from MnO toward the end of the series. As for the other oxides, the ground state is AFM, and in PBE0, it is separated by 0.27 eV from the FM solution.

The nature of the gap in NiO has been widely debated, and it is generally accepted that it has mixed charge transfer and Mott–Hubbard character, as described in the Zaanen–Sawatzky–Allen model.^23^ At the PBE0 level, and optimizing both atomic positions and lattice constants, NiO exhibits an indirect band gap of 5.30 eV and a direct gap of 6.44 eV, and a dielectric constant ε_∞_ = 4.75, Table 8.

**Table 8:** KS-DFT Indirect (*E*_g_^i^) and Direct (*E*_g_^d^) Band Gaps (in Electronvolts), Magnetic Moment (μ_B_/atom), and Dielectric Constant for NiO

NiO	*E*_g_^i^ (eV)	*E*_g_^d^ (eV)	*M* (μ_B_/atom)	ε_∞_
PBE0 (α = 0.25)	5.30	6.44	1.71	4.75
PBE0_DD_ (α = 1/sc – ε_∞_)	4.47	5.62	1.66	5.49
experiment			1.90^a^^,^^b^	5.76^c^
XAS + XES	4.0^d^			
PES + BIS	4.3^e^			
optical absorption		3.7^f^, 3.87^g^		

a See ref (89).

b See ref (96).

c See ref (98).

d See ref (29).

e See ref (100).

f See ref (30).

g See ref (101).

At the PBE0_DD_ level, we obtain a self-consistent value of ε_∞_ = 5.49 corresponding to α = 0.182. *E*_g_^i^ becomes 4.47 eV and *E*_g_^d^ 5.62 eV (Table 8). A small change of 0.05 μ_B_/atom is observed in magnetization.

At the PBE0_exp_ level (where α = 0.174 is obtained as the inverse of the experimental dielectric constant, 5.76),^98^ the gap values are about 0.1–0.2 eV smaller than those in PBE0_DD_ (Table S2).

Experimentally, band gap values in the range of 3.7–4.3 eV have been reported (Table 8), with optical absorption measurements giving smaller gaps than those derived from photoemission or X-ray absorption.^84^ We note that while the PBE0 functional fails in giving a reasonable estimate of the indirect band gap, with errors of about 22%, the use of dielectric-dependent functionals provides much better estimates of this quantity (errors of around 7%, Table 8). The direct band gap is instead always significantly overestimated by 1.8 eV (PBE0_DD_) and 2.6 eV (PBE0), with errors around 33–41%.

We move now to the results of the CTLs (Table 9). In the positively charged 4 × 4 × 4 supercell, there is a Ni ion with a magnetic moment of 0.74 μ_B_/atom, i.e., 0.92 μ_B_/atom lower than in the ground state. This shows that the hole forms in the 3d shell and the Ni ion formally goes from Ni^2+^(3d^8^) to Ni^3+^(3d^7^). In fact, the Ni 3d levels are hybridized with the O 2p ones, as shown by the DOS curves, and the state is not a purely Ni 3d state (see Figure S1d in the SI). The addition of an excess electron, however, always results in a completely delocalized solution, with the added electron redistributed over all of the Ni atoms of the supercell. The attempts to favor the formation of a polaron associated with the localized charge in the solid failed.

**Table 9:** Indirect (*E*_g_^i^) and Direct (*E*_g_^d^) Band Gap (in Electronvolts) of NiO for Supercells of Increasing Size Computed According to the CTLs Method

cell size	2 × 1 × 1	2 × 2 × 2	4 × 3 × 2	4 × 3 × 3	4 × 4 × 4
no. of atoms	4	16	48	72	128
*E*_g_^i^	2.87	3.41	3.67	3.65	3.79
*E*_g_^d^	3.16	4.24	4.18	4.12	4.17

**Figure 4:**
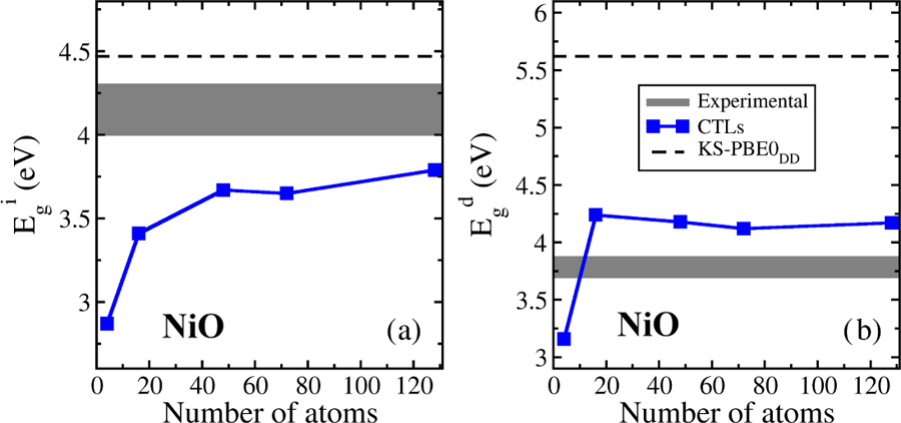
(a) Indirect and (b) direct band gaps of NiO computed with the CTL approach as a function of the supercell size. The black dashed lines represent the indirect and direct KS-PBE0_DD_ band gap, and the gray interval represents the range of experimental values.

The band gap data start to become reliable from the 2 × 2 × 2 supercell (16 atoms, Table 9 and Figure 4). The indirect band gap, *E*_g_^i^, increases with the cell size and reaches 3.6–3.8 eV when the cell contains more than 50 atoms. For instance, we obtain 3.79 eV with the largest supercell. In a similar way, rather stable values are obtained for *E*_g_^d^, with 4.17 eV being the best estimate (largest cell, Table 9).

The indirect gap computed with CTLs (3.79 eV) is thus smaller than that computed at the PBE0_DD_ level (4.47 eV, Table 8) and not too far from the experiment which reports an indirect band gap between 4 and 4.3 eV.^29,100^

The direct band gap computed with CTLs (*E*_g_^d^ = 4.17 eV) is much smaller than that computed at the KS-PBE0_DD_ level (5.62 eV) and much closer to the experimental measures of the direct band gap (3.7–3.9 eV).^30,101^ In this respect, the gap computed with the CTLs procedure shows an overall better agreement with experiment compared to the KS approach. As for CoO, we observed a significant energy contribution (around 0.64 eV) arising from relaxation of the charged cells and consequent localization of electrons and holes (Figure S3 in the SI, blue diamonds).

### 3.6 CuO

CuO is the last TM oxide considered. Here, Cu is Cu^2+^(3d^9^). In an octahedral field, this would result in a (*t*_2g_)^6^(*e*_g_)^3^ configuration, which is Jahn–Teller distorted so that the unit cell is no longer cubic but becomes monoclinic (*C*2/*c* structure). In an ionic picture, each Cu should have one unpaired electron. We compute a magnetization of 0.72 μ_B_ per Cu ion, which means that the material has a substantial covalent contribution, 28%, the largest value found so far. We used a double cell to compare the relative stabilities of the AFM and FM solutions. In CuO, there are four different AFM configurations: AFM1 (↑↑↓↓), AFM2 (↑↓↑↓), AFM3 (↓↑↑↓), and AFM4 (↑↓↓↑), all quite close in energy, but the last one, AFM4, is the most stable and is 0.10 eV lower in energy than the FM configuration.

At the PBE0 level, CuO exhibits an indirect KS band gap of 3.48 eV and a direct gap of 4.37 eV (optimized geometry, Table 10) (dielectric constant ε_∞_ = 5.05). Differently from the previous systems, non-negligible changes on the band gap are observed if the calculations are done using the experimental geometry (Table S2). The band gap is reduced by about 0.3 eV, showing that this property is more sensitive to structural changes than the magnetization or the dielectric constant. This is due to the deviation of the PBE0 lattice parameters compared to experiment: *a*, *b*, *c*, and β changed from 4.653 Å, 3.410 Å, 5.108 Å, and 90.48° (exp.)^99^ to 4.839 Å, 3.199 Å, 5.018 Å, and 102.73° (PBE0), respectively.

**Table 10:** KS-DFT Indirect (*E*_g_^i^) and Direct (*E*_g_^d^) Band Gaps (in Electronvolts), Magnetic Moment (μ_B_/atom), and Dielectric Constant for CuO

CuO	*E*_g_^i^ (eV)	*E*_g_^d^ (eV)	*M* (μ_B_/atom)	ε_∞_
PBE0 (α = 0.25)	3.48	4.37	0.72	5.05
PBE0_DD_ (α = 1/sc – ε_∞_)	2.29	3.18	0.66	5.74
experiment			0.69^a^	6.46^b^
XPS + BIS	1.4–1.7^c^			
optical absorption		1.44^d^, 1.57^e^		

a See ref (102).

b See ref (86).

c See ref (103).

d See ref (104).

e See ref (105).

For the PBE0_DD_ calculations, we obtain ε_∞_ = 5.74, not far from the experimental value, 6.46^86^ (Table 10). This corresponds to an α value of 0.174, which provides PBE0_DD_*E*_g_^i^ = 2.29 eV and *E*_g_^d^ = 3.18 eV (Table 10). A small reduction of 0.06 μ_B_/atom occurs in the magnetization with respect to PBE0.

These values can now be compared to the experimentally reported band gaps, which are in the range of 1.4–1.7 eV (Table 10).

**Figure 5:**
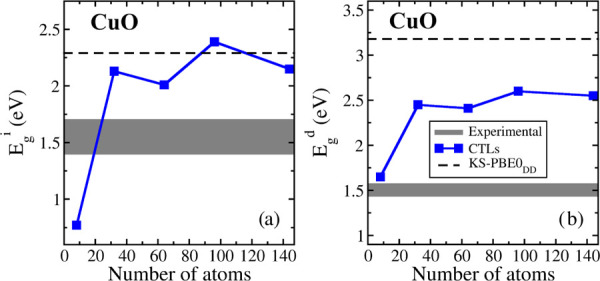
(a) Indirect and (b) direct band gaps of CuO computed with the CTLs approach as a function of the supercell size. The black dashed lines represent the indirect and direct KS-PBE0_DD_ band gap, and the gray interval represents the range of experimental values.

We now move to the CTLs results (Table 11 and Figure 5). Removing or adding one electron to the neutral cell (treated with PBE0_DD_) results in both hole and excess electron largely delocalized on the Cu 3d states. This is different from the previous cases and can be attributed to the higher degree of covalency in the material.

**Table 11:** Indirect (*E*_g_^i^) and Direct (*E*_g_^d^) Band Gap (in Electronvolts) of CuO for Supercells of Increasing Size Computed According to the CTL Method

cell size	1 × 1 × 1	2 × 2 × 1	2 × 2 × 2	2 × 3 × 2	3 × 3 × 2
no. of atoms	8	32	64	96	144
*E*_g_^i^	0.77	2.13	2.01	2.39	2.15
*E*_g_^d^	1.65	2.45	2.41	2.60	2.55

The small 2 × 2 × 1 supercell containing 32 atoms provides direct and indirect gaps, 2.13 and 2.45 eV, respectively, which are quite close to those obtained with the largest supercell (Table 11 and Figure 5).

The indirect band gap, *E*_g_^i^, becomes 2.15 eV in the 3 × 3 × 2 supercell; the value seems to be reasonably converged (within roughly 0.2 eV). Note that this value is quite close to the KS indirect band gap computed at the PBE0_DD_ level (2.29 eV, Table 10).

The best estimate of the indirect band gaps with the CTLs (2.15 eV) is larger than the experimental values (1.4–1.7 eV) but in better agreement than the KS band gap at the PBE0 or PBE0_DD_ levels, 3.48 and 2.29 eV, respectively. Similarly, the best estimate of the direct band gap with CTLs (2.55 eV) is significantly closer to the experimental range (1.4–1.6 eV) than KS band gaps at the PBE0 or PBE0_DD_ levels, 4.37 and 3.18 eV, respectively. Once more, CTLs provide better values than the KS-DFT approach. As for previous cases (MnO and Cu_2_O), relaxation effects on large charged cells are small, around 0.09 eV (Figure S3 in the SI (green triangle up)).

## 4 Discussion and Conclusions

In this work, we have addressed the complex nature of transition-metal oxides with magnetic character and their band gap. MnO, CoO, NiO, and CuO have been studied with both hybrid functional calculations and with an alternative procedure based on the charge transition levels. Cu_2_O has also been considered as a test case of nonmagnetic oxide. The method is based on the use of charge transition levels (CTLs), and the band gap is estimated from energy levels of the material with one electron added and one electron removed. FeO has been studied only at the hybrid functional level since unsurmountable convergence problems have been encountered in the calculation of CTLs for this oxide. The idea behind the CTL approach to the calculation of band gaps is that some oxides have very localized 3d states and can be described with the Hubbard model. The approach, however, is fully ab initio, and the nature of the final wave function for the generated electron and hole is determined self-consistently and can be localized or delocalized.

This work has two objectives: first, a completely different way to compute the band gap of a highly correlated material is proposed, based on the calculation of energy levels of charged states; in this respect, the study provides a test of the validity of the KS approach for the calculation of the band gap of this class of highly correlated solids (all display indirect band gap with exception of Cu_2_O). Second, the analysis of the electron and spin densities of the system with one electron added and one electron removed allows one to better define the nature of the fundamental electronic transition. The results show that this is related to the excitation from a more or less localized filled 3d level of a TM ion to the empty 3d states of neighboring TM ions, depending on the Mott–Hubbard vs charge transfer nature of the material.

The method proposed is parameter-free. If one uses a dielectric-dependent hybrid functional as a starting point, the amount of Fock exchange to be used (α) can be deduced from the dielectric constant of the material (α = 1/ε), eliminating the necessity to tune the α value against experimental values or the problems caused by a fixed value of this parameter. However, the approach is general and any hybrid functional can be used as a starting point for the calculation of the band gap with CTLs.

With this fully ab initio approach, which takes into account effects induced by extra electrons/holes in the system, we computed direct and indirect band gaps for the series of oxides mentioned above. The results have been checked versus supercell size.

**Figure 6:**
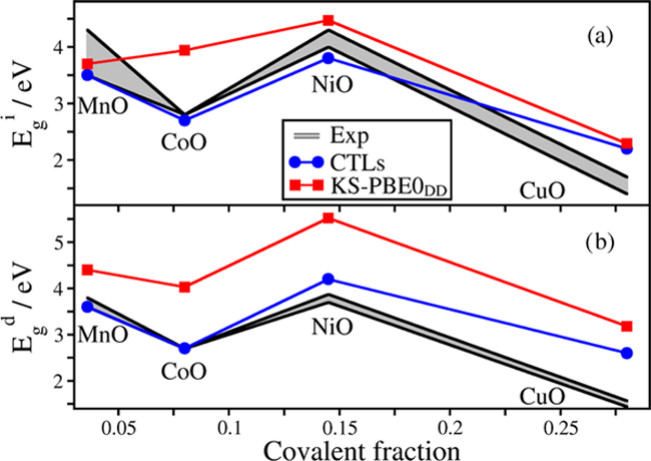
Trend of band gaps as a function of the covalent fraction of the oxides. (a) Indirect band gaps and (b) direct band gaps. The gray intervals indicate the range of experimental values, the blue dots are for band gaps calculated via CTLs, and the red squares are for KS band gaps.

Figure 6 shows the band gaps as a function of the covalent fraction of the oxides considered. As already pointed out above, when moving to the right of the periodic table, we find a systematic increase of the covalent character.

In general, the indirect band gaps computed with the CTLs are slightly better than those obtained with a hybrid functional (Figure 6a). This shows that the use of KS-DFT in combination with hybrid functionals for the study of highly correlated oxides provides sufficiently accurate results, despite the intrinsic limitations present in the KS estimate of band gaps. On the other hand, we have found a clear improvement in the direct band gap estimate (Figure 6b). In all cases considered, the band gap obtained with CTLs is found to be in better agreement with the experiment than the KS results. In some cases, e.g., CoO and NiO, the improvement is substantial. Upon full relaxation of charged supercells, we found that for some oxides, relaxation is small, while for other oxides, as NiO and CoO, we find substantial deviations due to polaron formation.

We now compare the CTL results to calculations performed with the state-of-the-art screened hybrid functional for solids, HSE06.^106^ We first observed that our calculations with HSE06 are generally close to those reported in ref (27). We see that in some cases such as FeO and MnO (direct gap), HSE06 performs better than PBE0 in reproducing the band gap, while a general tendency toward the overestimation can be observed. HSE06_DD_ mitigates this tendency, but CoO and NiO, for instance, remain a critical case. It is worth noting, in this respect, that the results obtained with CTLs result are more robust over the whole series of magnetic insulators.

When we compare the results of the CTLs with other methods such as the GW approach (Table 12), we find a general good performance of CTLs. In particular, the gap computed with CTLs shows a comparable or even better performance compared to G_0_W_0_@HSE03, G_0_W_0_@HSE06, GW^RPA^, or GW^LF^ + V_d_ calculations reported in the literature^27,40,41,45^ (Table 12).

**Table 12:** Indirect (*E*_g_^i^) and Direct (*E*_g_^d^) Band Gaps (in Electronvolts), from Different Theoretical Approaches and Experimental Measurements

	MnO		FeO		CoO		NiO		CuO		Cu_2_O
method	*E*_g_^i^	*E*_g_^d^	*E*_g_^i^	*E*_g_^d^	*E*_g_^i^	*E*_g_^d^	*E*_g_^i^	*E*_g_^d^	*E*_g_^i^	*E*_g_^d^	*E*_g_^d^
This Work											
CTL	3.52	3.64			2.66	2.71	3.79	4.17	2.15	2.55	2.55
HSE06	3.19	3.87	2.16	2.43	3.99	4.02	4.83	5.72	2.78	3.63	2.30
HSE06_DD_	3.06	3.71			3.30	3.35	4.07	5.11	1.80	2.66	2.26
Previous Works											
HSE06^a^	2.92	3.56	2.21	2.66	3.50	4.29	4.56	5.06			
HSE06^b^	4.77		2.41		2.82		4.09				
HSE06^c^									2.74	3.26	2.02
G_0_W_0_@HSE03^d^	3.4	4.0	2.2	2.3	3.4	4.5	4.7	5.2			
G_0_W_0_@HSE06^e^									3.6	3.5	
GW^RPA^ [f]^f^	3.81		1.65		3.23		4.28		2.49		1.59
GW^LF^ + V_d_ [f]	3.36		2.14		2.80		3.48		1.19		2.03
Experiment											
PES + BIS	3.9 ± 0.4^g^				2.5 ± 0.3^h^		4.3^i^		1.4–1.7^j^		
XAS + XES	4.1^k^				2.6^k^		4.0^k^				
OA		3.6–3.8^l^		2.40^m^		2.7^n^		3.7^n^		1.44^o^	2.17
								3.87^p^		1.57^q^	2.62^r^

a See ref (27).

b See ref (106).

c See ref (107).

d See ref (40).

e See ref (41).

f See ref (45).

g See ref (90).

h See ref (28).

i See ref (100).

j See ref (103).

k See ref (29).

l See ref (91).

m See ref (95).

n See ref (30).

o See ref (104).

p See ref (101).

q See ref (105).

r See ref (87).

In fact, the approach is not free from limitations. The most severe one is that the results need to be checked versus cell size to obtain converged values. In this respect, the use of semilocal or short-range hybrid functionals could mitigate the problem. However, hybrid functionals represent a notable step forward with respect to the semilocal ones when one deals with localized states in a CTL approach.^53^

In conclusion, this work provides an alternative approach for the determination of the band gap of magnetic insulators that goes beyond the KS approximation. Furthermore, the method allows an accurate description of the nature of the transition responsible for the excitation which is not always easy to obtain from the analysis of the DOS curves based on the KS levels.

## Supporting Information Available

Basis set effect on the antiferromagnetic (AFM) solution of NiO; ground-state properties for the AFM solution of all of the materials considered (MnO, FeO, CoO, NiO, CuO, Cu_2_O) at the experimental cell parameters; density of states (DOS) for the AFM solution of monoxides at the PBE0_DD_ level; and computed thermodynamic band gaps and the energy contribution due to the structural relaxation in the supercells (PDF)
