# Supplementary material for: Band Gap in Magnetic Insulators from a Charge Transition Level Approach
Source: J Chem Theory Comput. 2020 May 19;16(6):3786–98. doi: 10.1021/acs.jctc.0c00134 (PMC8007096; doi:10.1021/acs.jctc.0c00134)
Supplement: Supplementary file 1 — ct0c00134_si_001.pdf [file ct0c00134_si_001.pdf]

Supporting Information: Band Gap in Magnetic Insulators from a Charge Transition Levels Approach

Luis A. Cipriano, Giovanni Di Liberto, Sergio Tosoni, and Gianfranco Pacchioni

Dipartimento di Scienza dei Materiali, Università di Milano–Bicocca, via R. Cozzi 55, 20125 Milano, Italy

1. Basis Set Effect in the Antiferromagnetic (AFM) Solution of NiO

The effect of the basis set on the electronic structure of NiO (in the antiferromagnetic configuration, AFM) has been evaluated with the CRYSTAL17 code,[1] comparing all electron Pople basis set[2] and Alhrichs-type basis set (Pob-TZVP).[3] Direct (E_g^d) and indirect (E_g^i) Kohn-Sham (KS-DFT) band gaps (in Electronvolts), as well as the magnetic moment (in μ_B*/atom)* and the dielectric constant are reported in Table S1, using both the PBE0 functional[4,5] and its dielectric-dependent formulation ($PBE0_{DD}$). Calculations are done both at the experimental ionic structure (referred to as Exp in Table S1, $Fm\bar{3}m$ space group, a = 4.177 Å)[6] and on a fully relaxed structure (Opt in Table S1).

Table S1. Effect of the basis set in the KS-DFT indirect (E_g^i) and direct (E_g^d) band gaps (in Electronvols), magnetic moment (μ_B/atom), and dielectric constant for NiO.

NiO	Geom	E_g^i (eV)	E_g^d (eV)	M ($\mu_B/atom$)	Dielectric constant	Basis set
PBE0(α=0.25)	Opt	5.30	6.44	1.71	4.75	
PBE0(α=0.25)	Exp	5.30	6.42	1.71	4.75	Ahlrichs
$PBE0_{DD}$ ($\alpha=1/sc\text{-}\varepsilon_\infty$)	Opt	4.47	5.62	1.66	5.49	
$PBE0_{exp}$ ($\alpha=1/\varepsilon_\infty^{exp}$)	Exp	4.26	5.52	1.66	5.61	
PBE0 (α=0.25)	Opt	5.65	6.45	1.73	3.78	
PBE0 (α=0.25)	Exp	5.79	6.61	1.74	3.68	Pople
$PBE0_{DD}$ ($\alpha=1/sc\text{-}\varepsilon_\infty$)	Opt	5.95	6.80	1.75	3. 59	
$PBE0_{exp}$ ($\alpha=1/\varepsilon_\infty^{exp}$)	Exp	4.68	5.53	1.68	4.55	
Experiment				1.90[a,b]	5.76[c]	
XAS+XES		4.0[d]				
PES+BIS		4.3[e]				
Optical absorption			3.7[f], 3.87[g]			

[a]See ref 7. [b]See ref 8. [c]See ref 9. [d]See ref 10. [e]See ref 11. [f]See ref 12. [g]See ref 13.

From Table S1, we concluded that the Pob-TZVP basis set provides a general better accuracy, in particular in the calculation of the dielectric constant. For this reason, we rely on this type of basis set for all calculations reported in the present work.

2. Ground State Properties for the AFM Configuration with the Experimental Cell Parameters

The ground state properties for each MT monoxide using the experimental cell parameters and the Pob-TZVP basis set have been done at PBE0 and $PBE0_{exp}$ ($\alpha=1/\varepsilon_\infty^{exp}$) level. $PBE0_{exp}$ means that the experimental dielectric constant was used to determine the exact Fock exchange in the PBE0 functional. The average experimental dielectric constant for FeO was calculated from the two reported values, 9.24,[14] and 11.1.[15] The results are shown following in the Table S2.

Table S2. Ground states properties using the experimental cell parameters at PBE0 and $PBE0_{exp}$ ($\alpha=1/\varepsilon_\infty^{exp}$) level.

TM oxide	Method	E_g^i (eV)	E_g^d (eV)	M (μ_B/*atom*)	Dielectric constant
MnO	PBE0 (α=0.25)	3.91	4.59	4.82	4.25
	$PBE0_{exp}$ ($\alpha=1/\varepsilon_\infty^{exp}$)	3.37	4.04	4.80	4.50
FeO	PBE0 (α=0.25)	2.12	2.18	3.74	---
	$PBE0_{exp}$ ($\alpha=1/\varepsilon_\infty^{exp}$)	1.22	1.29	3.72	---
CoO	PBE0 (α=0.25)	4.69	4.85	2.76	4.54
	$PBE0_{exp}$ ($\alpha=1/\varepsilon_\infty^{exp}$)	3.57	3.70	2.72	5.08
NiO	PBE0 (α=0.25)	5.30	6.42	1.71	4.75
	$PBE0_{exp}$ ($\alpha=1/\varepsilon_\infty^{exp}$)	4.26	5.52	1.66	5.61
CuO	PBE0 (α=0.25)	3.20	4.08	0.71	4.74
	$PBE0_{exp}$ ($\alpha=1/\varepsilon_\infty^{exp}$)	1.73	2.85	0.64	6.13
Cu_2O	PBE0 (α=0.25)	---	2.54	---	3.72
	$PBE0_{exp}$ ($\alpha=1/\varepsilon_\infty^{exp}$)	---	2.05	---	4.52

3. Density of States (DOS) for the AFM Solution of Monoxides at $PBE0_{DD}$ Level

The Density of States (DOS, Fig. S1) has been calculated with $PBE0_{DD}$/pob-TZVP using the 2×1×1 supercell, which have two TM oxides (Mn, Fe, Co, Ni, and Cu) and two O atoms, this in order to account the antiferromagnetic ordering of these oxides. A fine grid (corresponding to a shrinking factor 10 in the Pack-Monkhorst scheme) has been adopted to sample the reciprocal space.

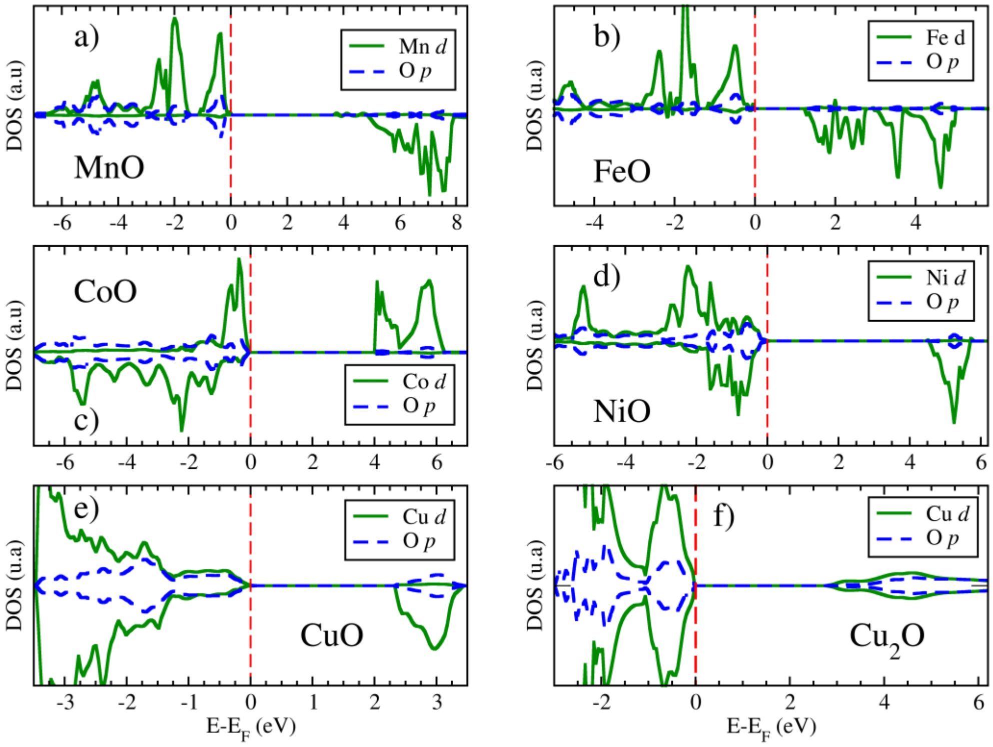

Figure S1. Density of States for: a) MnO, b) FeO, c) CoO, d) NiO, e) CuO, and f) Cu_2O at $PBE0_{DD}$ level, for FeO was considering the average experimental dielectric constant. Green line and blue dotted line represent the *d* states for the cation (Mn, Fe, Co, Ni, Cu) and *p* states for O, respectively. The red dotted line represents the Fermi level.

4. Thermodynamic Band Gaps

Optical transition levels (ϵ^{opt}), as stated above, do not include relaxation effects and can be directly compared with the position of the defect levels estimated from optical excitation; on the other hand, it can be interesting to estimate also the effects related to the geometrical relaxation upon trapping of charged species[16,17] and obtain a thermodynamic estimate of the band gap. Thus, we define

$$\epsilon^{therm}(+1/0) = \epsilon^{opt}(+1/0) + E_{rel}(+1/0) \quad (1)$$

$$\epsilon^{therm}(0/-1) = \epsilon^{opt}(0/-1) - E_{rel}(0/-1) \quad (2)$$

Where E_{rel} is defined as the absolute value of the difference between the relaxed configuration and the energy of the first SCF.[18,19] The thermodynamic band gap, E_g *(therm)*, is computed as:

$$E_g(therm) = \epsilon^{opt}(0/-1) - \epsilon^{opt}(+1/0) - E_{rel}(+1/0) - E_{rel}(0/-1) =$$
$$= E_g(opt) - [E_{rel}(+1/0) + E_{rel}(0/-1)] \quad (3)$$

Given that E_{rel} is by definition positive, $E_g(therm)$ is always smaller than $E_g(opt)$. In particular, the larger the relaxation energy associated to the trapping of one extra electron or extra hole, the smaller the thermodynamic gap compared to the electronic gap, as depicted in Fig. S2.

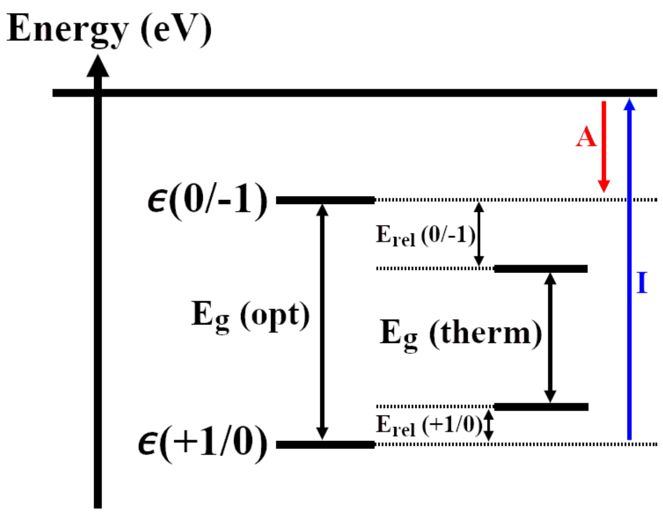

Figure S2. Graphical sketch of the energetics of the charge transition levels associated to the removal, $\epsilon^{opt}(+1/0)$, and addition, $\epsilon^{opt}(0/-1)$, of one electron. The reference vacuum level energy is set at 0 eV.

In Table S3, is shown the Indirect ($E_g{}^i$) and direct (($E_g{}^d$) thermodynamic band gaps of the system with primitive cubic cell (MnO, CoO, and NiO). While in Table S4, is show the values for CuO and Cu_2O cells.

Table S3. Indirect ($E_g{}^i$) and direct ($E_g{}^d$) thermodynamic band gap (in Electronvolts) of MnO, CoO, and NiO as a function of the cell size computed according to the CTLs method.

	Cell size	2×2×2	4×3×2	4×3×3	4×4×4
	n. of atoms	16	48	72	128
MnO	$E_g{}^i$ *(therm)*	NC	2.71	2.90	3.38
	$E_g{}^d$ *(therm)*	NC	3.04	3.22	3.50
CoO	$E_g{}^i$ *(therm)*	NC	1.40	2.17	1.79
	$E_g{}^d$ *(therm)*	NC	1.52	2.24	1.84
NiO	$E_g{}^i$ *(therm)*	0.60	2.39	2.78	2.93
	$E_g{}^d$ *(therm)*	1.43	2.90	3.25	3.31

Table S4. Indirect (E_g^i) and direct (E_g^d) thermodynamic band gap (in Electronvolts) of CuO and Cu_2O as a function of the cell size computed according to the CTLs method.

	Cell size	2×2×1	2×2×2	2×3×2	3×3×2
	n. of atoms	32	64	96	144
CuO	E_g^i *(therm)*	0.68	1.09	1.93	1.97
	E_g^d *(therm)*	1.00	1.49	2.14	2.37
	Cell size	2×2×1	2×2×2	3×2×2	3×3×2
	n. of atoms	24	48	72	108
Cu_2O	E_g^i *(therm)*	0.16	2.02	2.14	2.44
	E_g^d *(therm)*	0.84	2.38	2.86	2.99

5. Energies Contribution Due to Structural Relaxation in the Supercells

We have been relaxed the charge cells in oder to evaluate the impact of structural relaxation, finding small relaxation effects for the largest supercells of MnO, CuO and Cu_2O (around 0.03-0.11 eV), while for the largest supercells of CoO and NiO a larger relation effect was found (around 0.61-0.64 eV). In Fig. S3 is show the energy contribution due to the structural relaxation as a function of the supercell.

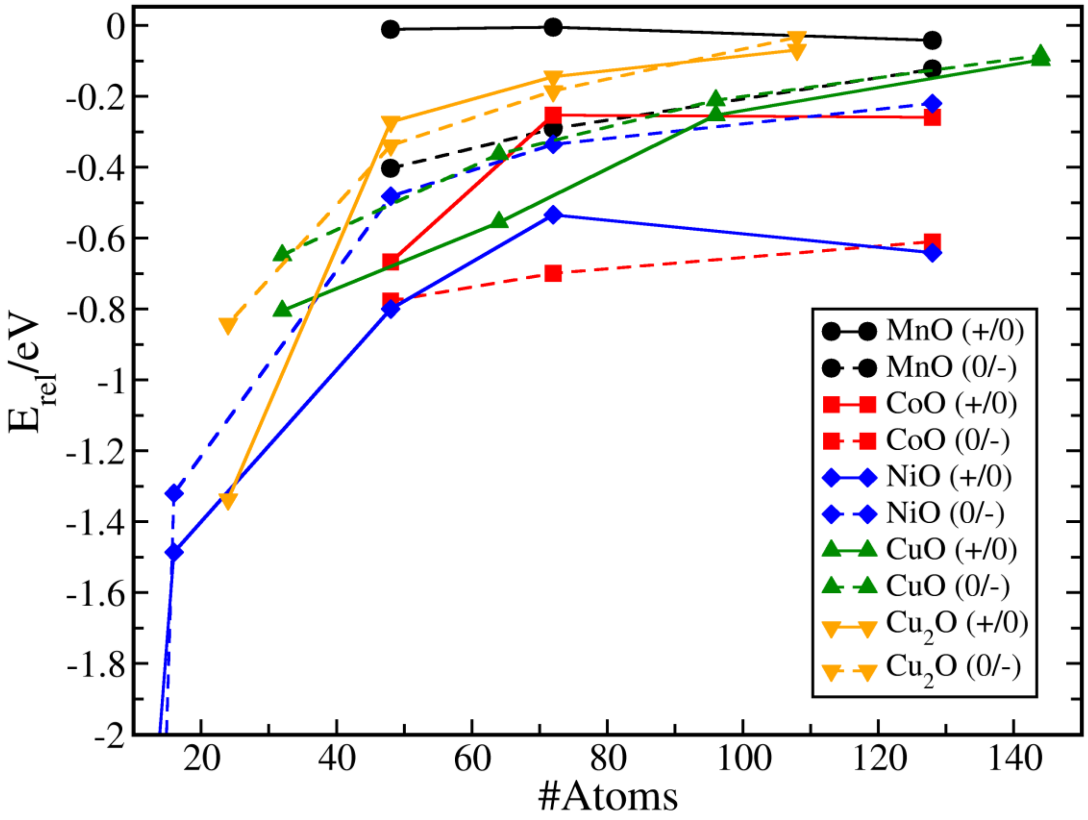

Figure S3. Energy relaxation as a function of the cell size. Black circles represent the MnO contribution, red squares the CoO contribution, blue diamonds the NiO contribution, green triangles up the CuO contribution, and orange triangles down the Cu_2O contribution with respect to the cell size.

References

(1) Dovesi, R.; Erba, A.; Orlando, R.; Zicovich-Wilson, C. M.; Civalleri, B.; Maschio, L.; Rérat, M.; Casassa, S.; Baima, J.; Salustro, S.; et al. Quantum-Mechanical Condensed Matter Simulations with CRYSTAL. *Wiley Interdiscip. Rev. Comput. Mol. Sci.* **2018**, *8* (4), e1360.

(2) Towler, M. D.; Allan, N. L.; Harrison, N. M.; Saunders, V. R.; Mackrodt, W. C.; Aprà, E. Ab Initio Study of MnO and NiO. *Phys. Rev. B* **1994**, *50* (8), 5041–5054.

(3) Peintinger, M. F.; Oliveira, D. V.; Bredow, T. Consistent Gaussian Basis Sets of Triple-Zeta Valence with Polarization Quality for Solid-State Calculations. *J. Comput. Chem.* **2013**, *34* (6), 451–459.

(4) Adamo, C.; Barone, V. Toward Reliable Density Functional Methods without

Adjustable Parameters: The PBE0 Model. *J. Chem. Phys.* **1999**, *110* (13), 6158–6170.

(5) Perdew, J. P.; Ernzerhof, M.; Burke, K. Rationale for Mixing Exact Exchange with Density Functional Approximations. *J. Chem. Phys.* **1996**, *105* (22), 9982–9985.

(6) Wyckoff, R. W. G. *The Structure of Cystals*; 1934.

(7) Cheetham, A. K.; Hope, D. A. O. Magnetic Ordering and Exchange Effects in the Antiferromagnetic Solid Solutions $Mn_xNi_{1-x}O$. *Phys. Rev. B* **1983**, *27* (11), 6964–6967.

(8) Roth, W. L. Magnetic Structures of MnO, FeO, CoO, and NiO. *Phys. Rev.* **1958**, *110* (6), 1333–1341.

(9) Rao, K. V; Smakula, A. Dielectric Properties of Cobalt Oxide, Nickel Oxide, and Their Mixed Crystals. *J. Appl. Phys.* **1965**, *36* (6), 2031–2038.

(10) Kurmaev, E. Z.; Wilks, R. G.; Moewes, A.; Finkelstein, L. D.; Shamin, S. N.; Kuneš, J. Oxygen X-Ray Emission and Absorption Spectra as a Probe of the Electronic Structure of Strongly Correlated Oxides. *Phys. Rev. B* **2008**, *77* (16), 165127.

(11) Sawatzky, G. A.; Allen, J. W. Magnitude and Origin of the Band Gap in NiO. *Phys. Rev. Lett.* **1984**, *53* (24), 2339–2342.

(12) Powell, R. J.; Spicer, W. E. Optical Properties of NiO and CoO. *Phys. Rev. B* **1970**, *2* (6), 2182–2193.

(13) Kang, T. D.; Lee, H. S.; Lee, H. Optical Properties of Black NiO and CoO Single Crystals Studied with Spectroscopic Ellipsometry. *J. Korean Phy. Soc.* **2007**, *50* (3), 632.

(14) Kugel, G.; Carabatos, C.; Hennion, B.; Prevot, B.; Revcolevschi, A.; Tocchetti, D. Lattice Dynamics of Wustite (FeO). *Phys. Rev. B* **1977**, *16* (1), 378–385.

(15) Prévot, B.; Biellmann, J.; Meftah, M. F.; Sieskind, M. Infra-Red Reflectivity of Non-Stoichiometric Ferrous Oxide. *Phys. status solidi* **1977**, *40* (2), 503–510.

(16) Oba, F.; Togo, A.; Tanaka, I.; Paier, J.; Kresse, G. Defect Energetics in ZnO: A Hybrid Hartree-Fock Density Functional Study. *Phys. Rev. B* **2008**, *77* (24), 245202.

(17) Gallino, F.; Pacchioni, G.; Di Valentin, C. Transition Levels of Defect Centers in ZnO by Hybrid Functionals and Localized Basis Set Approach. *J. Chem. Phys.* **2010**, *133* (14), 144512.

(18) Scherz, U.; Scheffler, M. Chapter 1 Density-Functional Theory of Sp-Bonded Defects

in III/V Semiconductors. In *Semiconductors and Semimetals*; Weber, E. R., Ed.; Semiconductors and Semimetals; Elsevier, 1993; Vol. 38, pp 1–58.

(19) Hedström, M.; Schindlmayr, A.; Schwarz, G.; Scheffler, M. Quasiparticle Corrections to the Electronic Properties of Anion Vacancies at GaAs(110) and InP(110). *Phys. Rev. Lett.* **2006**, *97* (22), 226401.
